# Supplementary material for: Dynamic survival analysis: Modelling the hazard function via ordinary differential equations
Source: Stat Methods Med Res. 2024 Aug 20;33(10):1768–82. doi: 10.1177/09622802241268504 (PMC11577698; doi:10.1177/09622802241268504)
Supplement: sj-pdf-1-smm-10.1177_09622802241268504 - Supplemental material for Dynamic survival analysis: Modelling the hazard function via ordinary differential equations [file sj-pdf-1-smm-10.1177_09622802241268504.pdf]

Journal Title
XX(X):1–13

©The Author(s) 2016
Reprints and permission:
sagepub.co.uk/journalsPermissions.nav

DOI: 10.1177/ToBeAssigned
www.sagepub.com/
SAGE

Appendix: "Dynamic survival analysis: modelling the hazard function via ordinary differential equations"

J. Andres Christen[1] and F. Javier Rubio[2]

Keywords
Autonomous ODE, Hazard function, ODE solver, Ordinary differential equations.

Appendix

Marginal posteriors for real data applications

Jacobian for the Hazard-Response system of ODEs

The Jacobian for the Hazard-Response system of ODEs (7) is:

$$J_{HT}(t,h,q,H,\boldsymbol{\theta}) = \begin{pmatrix} \lambda - \dfrac{2\lambda h}{\kappa} - \alpha q & -\alpha h & 0 \\ -\alpha q & \beta - \dfrac{2\beta q}{\kappa} - \alpha h & 0 \\ 1 & 0 & 0 \end{pmatrix},$$

where $\boldsymbol{\theta} = (\lambda, \kappa, \alpha, \beta)$ are the model parameters.

[1]Department of Statistics, Centre for Research in Mathematics (CIMAT). Guanajuato, Mexico
[2]Department of Statistical Science, University College London. London, UK

Corresponding author:
F. Javier Rubio Department of Statistical Science, University College London. London, UK
Email: f.j.rubio@ucl.ac.uk

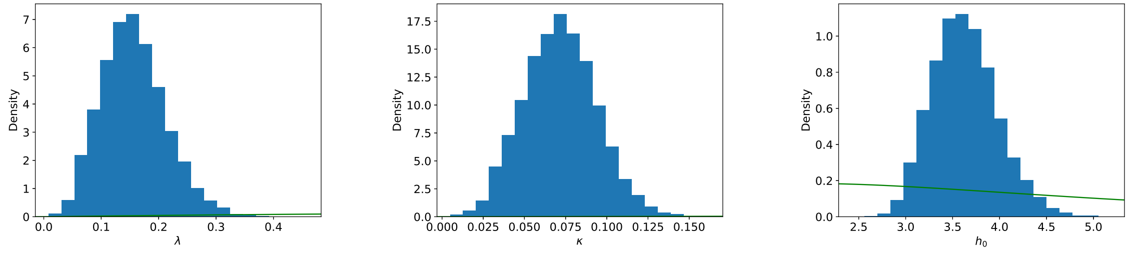

Figure 1. `LeukSurv` data: posterior distributions of the parameters of the logistic ODE model. (a) posterior of λ, (b) posterior of κ, and (c) posterior of h_0.

Logarithmic formulation of the hazard-response model

Define $\tilde{h}(t) = \log h(t)$ and $\tilde{q}(t) = \log q(t)$. Then, the hazard-response model (7) becomes

$$\begin{cases} \tilde{h}'(t) = \lambda \left(1 - \dfrac{\exp\left\{\tilde{h}(t)\right\}}{\kappa}\right) - \alpha \exp\left\{\tilde{q}(t)\right\}, & \tilde{h}(0) = \log\left\{h_0\right\} \\ \tilde{q}'(t) = \beta \left(1 - \dfrac{\exp\left\{\tilde{q}(t)\right\}}{\kappa}\right) - \alpha \exp\left\{\tilde{h}(t)\right\}, & \tilde{q}(0) = \log\left\{q_0\right\} \\ H'(t) = \exp\left\{\tilde{h}(t)\right\}, & H(0) = 0. \end{cases}$$

The corresponding Jacobian is:

$$J_{HT}(t, \tilde{h}, \tilde{q}, H, \boldsymbol{\theta}) = \begin{pmatrix} -\dfrac{\lambda}{\kappa} \exp\left\{\tilde{h}(t)\right\} & -\alpha \exp\left\{\tilde{q}(t)\right\} & 0 \\ -\alpha \exp\left\{\tilde{h}(t)\right\} & -\dfrac{\beta}{\kappa} \exp\left\{\tilde{q}(t)\right\} & 0 \\ \exp\left\{\tilde{h}(t)\right\} & 0 & 0 \end{pmatrix},$$

where $\boldsymbol{\theta} = (\lambda, \kappa, \alpha, \beta)$ are the model parameters.

A note on the posterior predictive hazard

Given $\pi(\boldsymbol{\theta} \mid \text{data})$ the posterior density for the parameter $\boldsymbol{\theta}$ of a hazard function $h_{\boldsymbol{\theta}}(t)$, one may calculate the posterior distribution of the hazard function, since at any t, $h(t \mid \boldsymbol{\theta}) = \frac{f(t|\boldsymbol{\theta})}{S(t|\boldsymbol{\theta})} \in \mathbb{R}^+$ and this is simply a function of the random variable $\boldsymbol{\theta}$. From a MCMC sample of the posterior, $\boldsymbol{\theta}^{(1)}, \ldots, \boldsymbol{\theta}^{(M)}$, one obtains a sample of the posterior hazard from $h(t \mid \boldsymbol{\theta}^{(1)}), \ldots, h(t \mid \boldsymbol{\theta}^{(M)})$.

On the other hand, we may calculate the predictive distribution of a hypothetical future $n+1$ patient

$$f_{t_{n+1}}(t \mid \text{data}) = \int_\Theta f(t \mid \boldsymbol{\theta}) \pi(\boldsymbol{\theta} \mid \text{data}) d\boldsymbol{\theta} = \int_\Theta h(t \mid \boldsymbol{\theta}) \exp(-H(t \mid \boldsymbol{\theta})) \pi(\boldsymbol{\theta} \mid \text{data}) d\boldsymbol{\theta}.$$

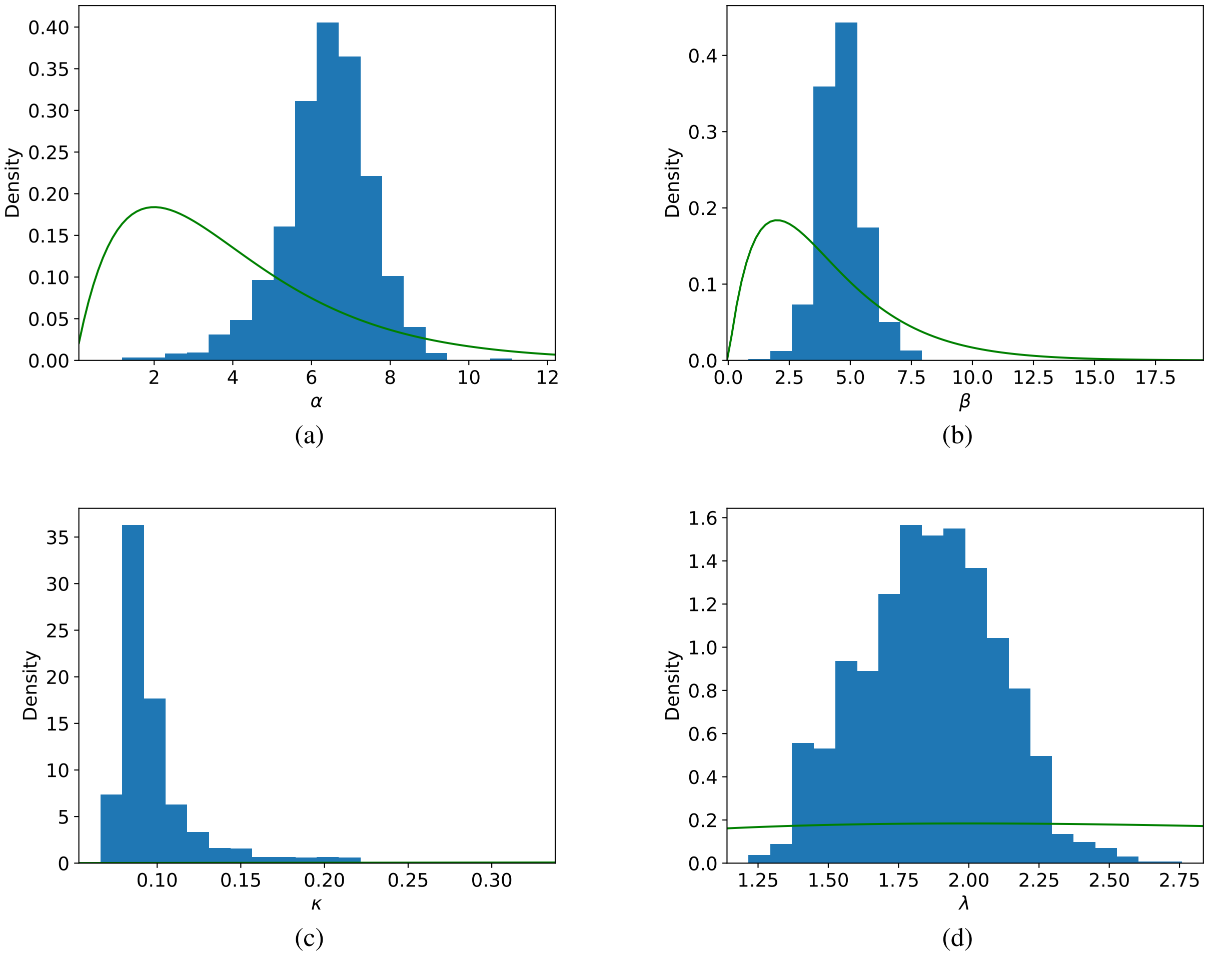

Figure 2. `rotterdam` data: Marginal posteriors for the Hazard-Response hazard function. The relevant part of the prior density is shown in green.

From this *fixed* posterior predictive density we may calculate its hazard function

$$h_{t_{n+1}}(t \mid \text{data}) = \frac{f_{t_{n+1}}(t \mid \text{data})}{1 - \int_0^t f_{t_{n+1}}(r \mid \text{data}) dr}.$$

This is the posterior predictive hazard and is the hazard to be analysed, if we wish to discuss the prognosis of a future patient, whereas the former represents the posterior uncertainty we have about the hazard in the population.

Unfortunately, besides the simplest cases, calculating $h_{t_{n+1}}(t \mid \text{data})$ in closed-form is not possible. However, we can obtain a Monte Carlo approximation using a posterior sample of the parameters, as follows

$$f_{t_{n+1}}(t \mid \text{data}) \approx \frac{1}{M}\sum_{j=1}^{M} h(t \mid \boldsymbol{\theta}^{(j)}) \exp\left\{-H(t \mid \boldsymbol{\theta}^{(j)})\right\}$$

and

$$1 - \int_0^t f_{t_{n+1}}(r \mid \text{data}) dr \approx \frac{1}{M}\sum_{j=1}^{M} \exp\left\{-H(t \mid \boldsymbol{\theta}^{(j)})\right\}.$$

Consequently

$$h_{t_{n+1}}(t \mid \text{data}) \approx \frac{\frac{1}{M}\sum_{j=1}^{M} h(t \mid \boldsymbol{\theta}^{(j)}) \exp\{-H(t \mid \boldsymbol{\theta}^{(j)})\}}{\frac{1}{M}\sum_{j=1}^{M} \exp\{-H(t \mid \boldsymbol{\theta}^{(j)})\}}.$$

Indeed, and by the same argument, the predictive survival function may be approximated by

$$S_{t_{n+1}}(t \mid \text{data}) \approx \frac{1}{M}\sum_{j=1}^{M} \exp\left\{-H(t \mid \boldsymbol{\theta}^{(j)})\right\}.$$

Simulation study results: Logistic ODE hazard model

Parameter	Mean	Median	SD	RMSE	coverage
		$n = 250$			
λ (0.5)	0.597	0.582	0.138	0.163	0.916
κ (0.05)	0.053	0.052	0.006	0.007	0.952
h_0 (3.5)	4.349	4.137	1.372	1.436	0.948
		$n = 500$			
λ (0.5)	0.545	0.538	0.089	0.100	0.932
κ (0.05)	0.051	0.051	0.004	0.004	0.936
h_0 (3.5)	3.976	3.863	0.914	0.983	0.932
		$n = 1000$			
λ (0.5)	0.527	0.523	0.061	0.070	0.924
κ (0.05)	0.051	0.051	0.003	0.003	0.944
h_0 (3.5)	3.754	3.697	0.617	0.656	0.932
		$n = 5000$			
λ (0.5)	0.506	0.505	0.026	0.027	0.936
κ (0.05)	0.050	0.050	0.001	0.001	0.932
h_0 (3.5)	3.530	3.519	0.259	0.265	0.944

Table 1. Simulation scenario 1 with 25% censoring rate. Average posterior mean, average posterior median, average posterior standard deviation, average RMSE with respect to the posterior mean, and coverage of the 95% credible intervals.

Parameter	Mean	Median	SD	RMSE	coverage
		$n = 250$			
λ (0.5)	0.704	0.679	0.237	0.298	0.920
κ (0.05)	0.059	0.059	0.013	0.014	0.936
h_0 (3.5)	4.448	4.226	1.431	1.501	0.936
		$n = 500$			
λ (0.5)	0.600	0.589	0.156	0.182	0.912
κ (0.05)	0.054	0.054	0.010	0.011	0.920
h_0 (3.5)	4.032	3.915	0.946	1.017	0.940
		$n = 1000$			
λ (0.5)	0.558	0.554	0.109	0.122	0.924
κ (0.05)	0.052	0.053	0.007	0.007	0.944
h_0 (3.5)	3.789	3.731	0.637	0.680	0.952
		$n = 5000$			
λ (0.5)	0.510	0.509	0.047	0.045	0.968
κ (0.05)	0.050	0.050	0.003	0.003	0.952
h_0 (3.5)	3.534	3.522	0.264	0.268	0.960

Table 2. Simulation scenario 1 with 50% censoring rate. Average posterior mean, average posterior median, average posterior standard deviation, average RMSE with respect to the posterior mean, and coverage of the 95% credible intervals.

Simulation study results: hazard-response model

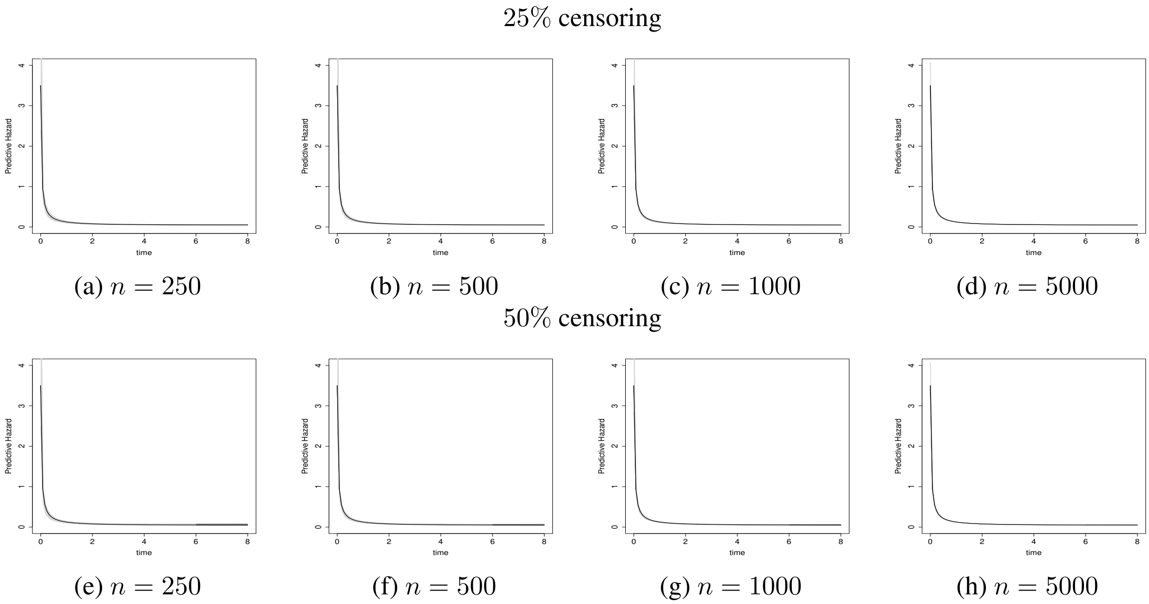

Figure 3. Simulation scenario 1: Predictive hazards and 95% predictive intervals. The dark gray area indicates the censoring point.

Parameter	Mean	Median	SD	RMSE	coverage
		$n = 250$			
λ (1.8)	2.446	1.755	0.850	1.041	0.965
κ (0.1)	0.141	0.082	0.076	0.088	0.985
α (6)	4.224	3.169	1.812	1.925	1.000
β (4.8)	4.824	3.567	2.171	0.674	1.000
		$n = 500$			
λ (1.8)	2.112	1.598	0.494	0.616	0.955
κ (0.1)	0.162	0.105	0.074	0.156	0.970
α (6)	4.297	3.334	1.615	1.954	0.965
β (4.8)	4.862	3.683	1.904	0.869	0.985
		$n = 1000$			
λ (1.8)	2.014	1.571	0.321	0.412	0.960
κ (0.1)	0.130	0.088	0.046	0.101	0.975
α (6)	4.665	3.755	1.426	1.646	0.975
β (4.8)	4.712	3.637	1.601	0.767	0.990
		$n = 5000$			
λ (1.8)	1.832	1.460	0.154	0.183	0.965
κ (0.1)	0.119	0.088	0.027	0.053	0.980
α (6)	5.303	4.326	0.745	1.053	0.950
β (4.8)	4.901	3.880	0.824	0.799	0.965

Table 3. Simulation scenario 2 with 25% censoring rate. Average posterior mean, average posterior median, average posterior standard deviation, average RMSE with respect to the posterior mean, and coverage of the 95% credible intervals.

Parameter	Mean	Median	SD	RMSE	coverage
			$n = 250$		
λ (1.8)	2.374	1.698	0.839	0.996	0.975
κ (0.1)	0.211	0.102	0.158	0.212	0.995
α (6)	3.989	2.921	1.848	2.130	0.990
β (4.8)	5.166	3.878	2.268	0.934	1.000
			$n = 500$		
λ (1.8)	2.098	1.580	0.510	0.594	0.985
κ (0.1)	0.183	0.100	0.112	0.176	0.990
α (6)	4.247	3.248	1.728	1.958	0.985
β (4.8)	5.005	3.814	1.986	0.756	1.000
			$n = 1000$		
λ (1.8)	1.999	1.559	0.328	0.399	0.970
κ (0.1)	0.147	0.093	0.069	0.134	0.990
α (6)	4.564	3.643	1.549	1.704	0.980
β (4.8)	4.863	3.735	1.694	0.747	1.000
			$n = 5000$		
λ (1.8)	1.824	1.454	0.157	0.188	0.950
κ (0.1)	0.127	0.090	0.035	0.072	0.960
α (6)	5.204	4.261	0.849	1.142	0.975
β (4.8)	4.963	3.926	0.856	0.847	0.955

Table 4. Simulation scenario 2 with 50% censoring rate. Average posterior mean, average posterior median, average posterior standard deviation, average RMSE with respect to the posterior mean, and coverage of the 95% credible intervals.

25% censoring

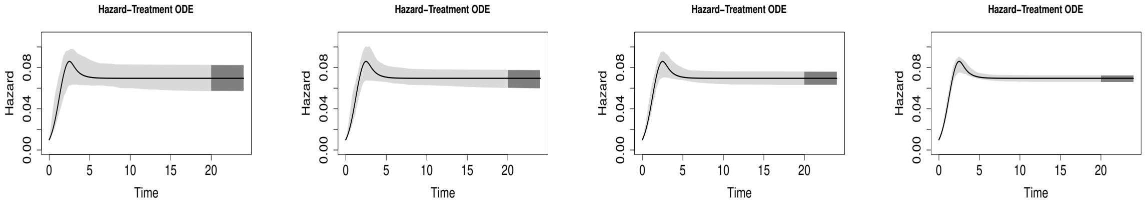

(a) $n = 250$ (b) $n = 500$ (c) $n = 1000$ (d) $n = 5000$

50% censoring

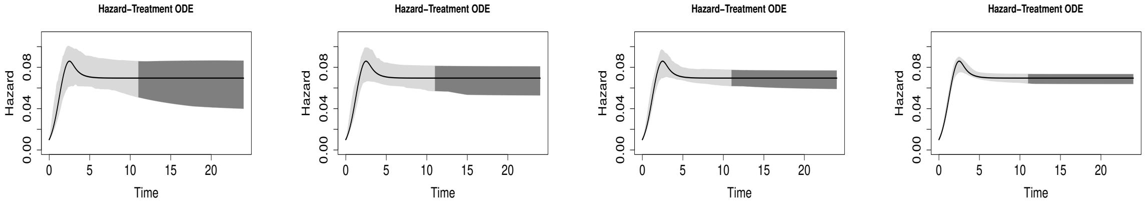

(e) $n = 250$ (f) $n = 500$ (g) $n = 1000$ (h) $n = 5000$

Figure 4. Simulation scenario 2: Predictive hazards and 95% predictive intervals. The dark gray area indicates the censoring point.

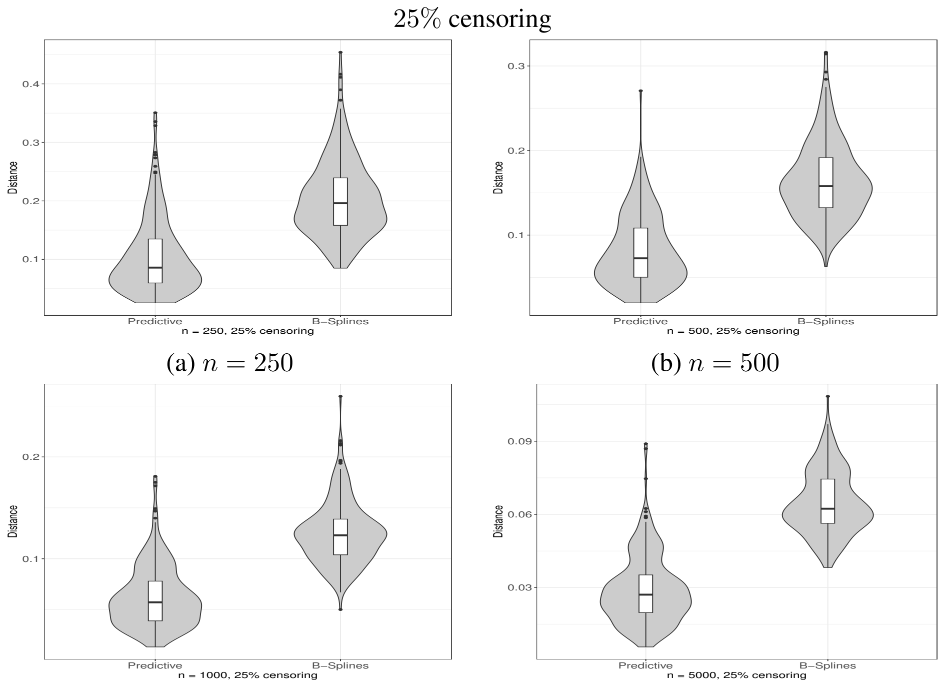

(a) $n = 250$ (b) $n = 500$

(c) $n = 1000$ (d) $n = 5000$

Figure 5. Simulation scenario 2, 25% censoring: Distance between the posterior predictive hazards and the B-Spline estimator to the true generating model.

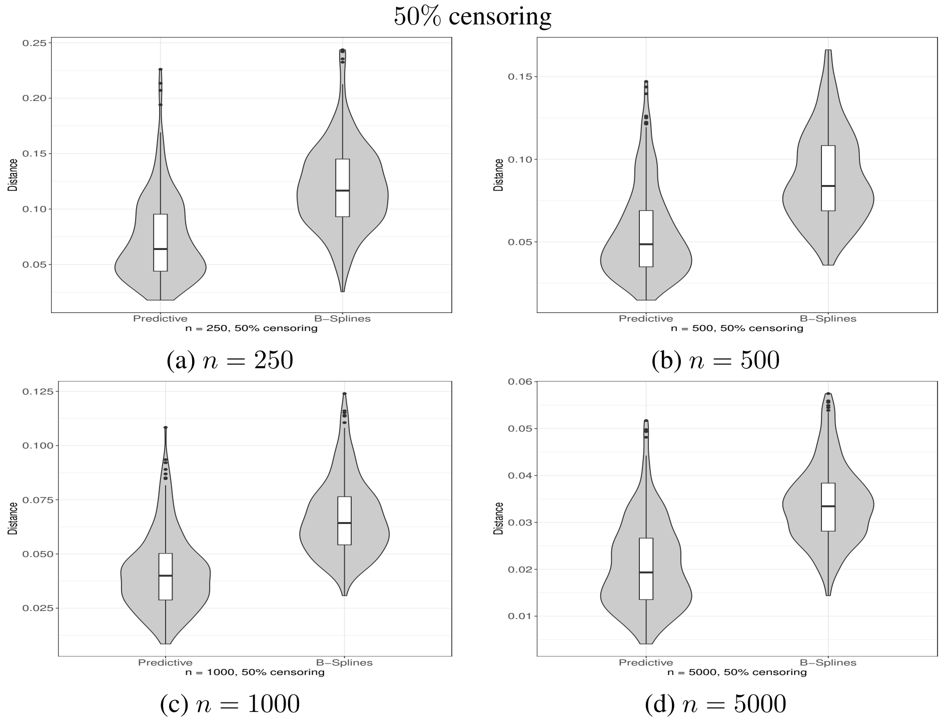

(a) $n = 250$ (b) $n = 500$

(c) $n = 1000$ (d) $n = 5000$

Figure 6. Simulation scenario 2, 50% censoring: Distance between the posterior predictive hazards and the B-Spline estimator to the true generating model.

References
